# *Nigella sativa* L. and Its Active Compound Thymoquinone in the Clinical Management of Diabetes: A Systematic Review

**DOI:** 10.3390/ijms232012111

**Published:** 2022-10-11

**Authors:** Mohamad Fawzi Mahomoodally, Muhammad Zakariyyah Aumeeruddy, Lesetja J. Legoabe, Domenico Montesano, Gokhan Zengin

**Affiliations:** 1 Institute of Research and Development, Duy Tan University, Da Nang 550000, Vietnam; 2 Faculty of Natural Sciences, Duy Tan University, Da Nang 550000, Vietnam; 3 Department of Health Sciences, Faculty of Medicine and Health Sciences, University of Mauritius, Réduit 230, Mauritius; 4 Centre of Excellence for Pharmaceutical Sciences (Pharmacen), North-West University, Mmabatho 2735, South Africa; 5 Department of Pharmacy, University of Naples Federico II, Via D. Montesano 49, 80131 Naples, Italy; 6 Department of Biology, Faculty of Science, Selcuk University, 42250 Konya, Turkey

**Keywords:** diabetes, *Nigella sativa*, thymoquinone, clinical studies

## Abstract

Despite existing conventional hypoglycemic drugs to manage diabetes, their non-availability and cost in low-income countries coupled with the associated side effects remain a major concern. Consequently, exploring for alternative treatments to manage diabetes has been a continuous priority. *Nigella sativa* L. (NS) (Family: Ranunculaceae) is regarded as a valuable traditional remedy in diabetes management and extensively studied for its biological properties. This systematic review provides a comprehensive and critical analysis of clinical studies on the efficacy, safety, and mechanism of action of NS and its compound thymoquinone (TQ) in diabetes management. The main scientific databases which were scrutinised were Scopus, PubMed, Google Scholar, and Web of Science. Data search was conducted from inception to January 2022. A total of 17 clinical studies were obtained; 16 studies on *Nigella sativa* L. and 1 study on its compound TQ. *N. sativa* was found to be highly potent in terms of its hypoglycemic activity when compared to placebo based on improvement in parameters including fasting blood glucose (FBG), postprandial blood glucose (PPBG), Hemoglobin A1C (HbA1c), homeostatic model assessment for insulin resistance (HOMA-IR), and homeostatic model assessment for assessment of beta-cell functionality (HOMA-β). The compound TQ in combination with a daily dose of metformin demonstrated a greater reduction in the levels of HbA1c and blood glucose compared to metformin alone. The bioavailability of TQ can be enhanced by using nanoparticulate drug delivery systems. Considering the findings of the clinical studies along with negligible adverse effects, NS has strong potential application in bioproduct development for the management of diabetes. Further investigations should explore the detailed mechanism of actions by which TQ exerts its therapeutic antidiabetic effects to provide more insights into its clinical use in the management of diabetes.

## 1. Introduction

Diabetes mellitus is a chronic condition in which the level of glucose in the blood is raised because the body cannot produce any or enough insulin or cannot make efficient use of the insulin it produces [1]. Type 1 diabetes (formerly called insulin-dependent, juvenile or childhood-onset diabetes) is characterised by a deficiency in insulin production in the body while type 2 diabetes (TD2), previously termed as non-insulin-dependent or adult-onset diabetes, is the most common type of diabetes, which accounts for around 90% of all diabetes worldwide [1,2]. Diabetes can lead to damage of the heart, blood vessels, eyes, kidneys, and nerves, and also increases the risk of heart disease and stroke [2]. The lack of access to affordable insulin remains a key barrier for successful treatment of diabetes and results in further complications and premature deaths. Besides availability, the cost of medications determines whether the treatment is affordable, e.g., 0.7% of households in high-income countries and 26.9% of households in low-income countries could not afford metformin. The lack of affordability of insulin was found to be higher with 2.8% and 63% of households in high-income and low-income countries, respectively, unable to afford it [1].

Recently, concerns about the associated side effects and discomfort of antidiabetic drugs have led to an increasing interest in the use of natural products. *Nigella sativa* L. (NS) seeds, also known as black seed (English), çörek otu (Turkish), habat-ul-sauda (Arabic) and kalonji in South Asia, are the black-coloured, funnel-shaped seeds of the *Nigella sativa* plant belonging to the Ranunculaceae family. The plant is cultivated in various regions such as Southern Europe, North Africa, Middle Eastern Mediterranean and the southern areas of Asia including Syria, Turkey, India, Pakistan, and Saudi Arabia. NS is considered a valuable traditional remedy in diabetes management (Table 1) and has been found to possess vast biological activities including antimicrobial, antioxidant, anti-inflammatory, anticancer, antidiabetic and cardioprotective properties amongst others [3]. 

Several reviews have previously been conducted on NS in the management of diabetes. For instance, in a review of clinical studies by Hamdan et al. [4] on a total of seven articles, NS was found to significantly reduce fasting blood glucose (FBG), 2 h postprandial blood glucose (PPBG), glycated hemoglobin A1C (HbA1c) and insulin resistance, while increasing serum insulin. In another review by Heshmati and Namazi [5], 19 eligible articles (2 human studies, 14 animal models and 3 in vivo/in vitro studies) were selected. NS modulated hyperglycemia and lipid profile dysfunction via several mechanisms including its antioxidant properties, effects on insulin secretion, glucose absorption, gluconeogenesis, and gene expression. Daryabeygi-Khotbehsara et al. [6] conducted a systematic review and meta-analysis of the effect of NS on glucose homeostasis and serum lipids in TD2. Analysis of seven studies revealed that NS significantly improved FBG [−17.84 mg/dL, 95% CI: −21.19 to −14.49] and HbA1c [−0.71%, 95% CI: −1.04 to −0.39]. Bule et al. [7] also carried out a systematic review and meta-analysis of the antidiabetic effect of thymoquinone (TQ) in animal studies. Nonetheless, a comprehensive review of the clinical efficacy of NS and TQ is lacking. The present review attempts to provide a comprehensive systematic review and critical analysis of clinical studies on the efficacy, safety, and mechanism of action of NS and its compound TQ in diabetes management.

## 2. Methods

### 2.1. Search Strategy 

The present systematic review was performed using the PRISMA guidelines. The following electronic databases were searched: Scopus, Google Scholar, PubMed, and Web of Science. Articles were retrieved from inception to January 2022. As for the keywords used, the word “*Nigella sativa*” was searched together with the following terms: “diabetes patient”, “diabetes trial”, “diabetes clinical”, “diabetes human studies”, and “diabetes randomised controlled trial”, “hypoglycemic patient”, “hypoglycemic trial”, “hypoglycemic clinical”, “hypoglycemic human studies”, and “hypoglycemic randomised controlled trial”. Similarly, besides the words “diabetes” and “hypoglycemic”, other associated terms were also used such as “fasting blood glucose”, “fasting blood sugar”, “glucose level”, “sugar level”, “blood glucose”, “HbA1c”, “glycated hemoglobin”, “glycosylated hemoglobin A”, “insulin level”, “insulin parameters”, and “Homeostatic Model Assessment (HOMA)”. Titles and abstracts were scanned on inclusion criteria. In addition, the reference lists of the retrieved articles were hand searched to find relevant studies.

### 2.2. Inclusion and Exclusion Criteria

The inclusion criteria were peer-reviewed articles published in the English language and studies comprising NS and TQ. Subjects in the clinical studies were Type 1 and/or type 2 diabetics, patients suffering from other conditions but whose diabetic parameters (e.g., glucose level) were tested, and also healthy subjects. Studies without a control group were also included. 

Articles were excluded by the following criteria: (1) review articles and case reports, (2) clinical studies investigating the antidiabetic activity of mixture and formulations, (3) studies that did not investigate the plant of interest, (4) studies not on hypoglycemic activity but on a condition associated with diabetes e.g., nephropathy, neuropathy, and retinopathy, (5) articles published in non-English language, (6) studies with abstract only and without full texts accessible, and (7) full-text articles without enough quantitative data, and (8) studies on glycemic index. 

### 2.3. Data Extraction

From the selected studies, the following data were extracted:

(a) General information: sample size, study design, country, and the year in which the study was conducted.
(b) Baseline characteristics of subjects: age, gender, and clinical data (healthy, diabetic patients, or patients suffering from other conditions).
(c) Intervention data: part used, administered dosages and administration frequency, duration of treatment, intake of oral hypoglycemic medications and/or insulin therapy during intervention.
(d) Results of studies including fasting blood glucose (FBG), hemoglobin A1c (HbA1c), postprandial blood glucose (PPBG), homeostatic model assessment for insulin resistance (HOMA-IR), homeostatic model assessment for beta-cell functionality (HOMA-β), homeostatic model assessment for insulin sensitivity (HOMA-S), and quantitative insulin-sensitivity check index (QUICKI). The mean difference for each parameter from baseline to end of the study was calculated.
(e) Any reported adverse effects.

### 2.4. Quality Assessment

The methodological quality of enrolled studies was assessed using the scoring system developed by Jadad et al. [25] where total score ranges from 0 to 5 points based on 5 criteria: (i) randomisation, (ii) suitable method of randomisation, (iii) blinding, (iv) suitable method of double blinding, and (v) withdrawals or drop-outs explanation. The scores of 3 or more represented high quality while 0–2 indicated a low-quality study. Articles were assessed by two independent reviewers. Any discrepancy was resolved by a third independent person. A meta-analysis could not be conducted to achieve profound statistical analysis since the majority of studies had missing data including lower bound and upper bound values of the difference between treatment and control, standard deviation of change, and *p*-value of difference. Consequently, meta-analysis was avoided to prevent any serious bias in the study results, and a quantitative report with a critical analysis of the included studies was performed. According to the Cochrane Handbook for Systematic Reviews of Interventions, “*A systematic review need not contain any meta-analyses…If there is considerable variation in results, and particularly if there is inconsistency in the direction of effect, it may be misleading to quote an average value for the intervention effect*.”, “*If studies are clinically diverse then a meta-analysis may be meaningless…Meta-analyses of studies that are at risk of bias may be seriously misleading. If bias is present in each (or some) of the individual studies, meta-analysis will simply compound the errors, and produce a ‘wrong’ result that may be interpreted as having more credibility. Finally, meta-analyses in the presence of serious publication and/or reporting biases are likely to produce an inappropriate summary.*” [26].

### 2.5. Study Selection

**Figure 1:**
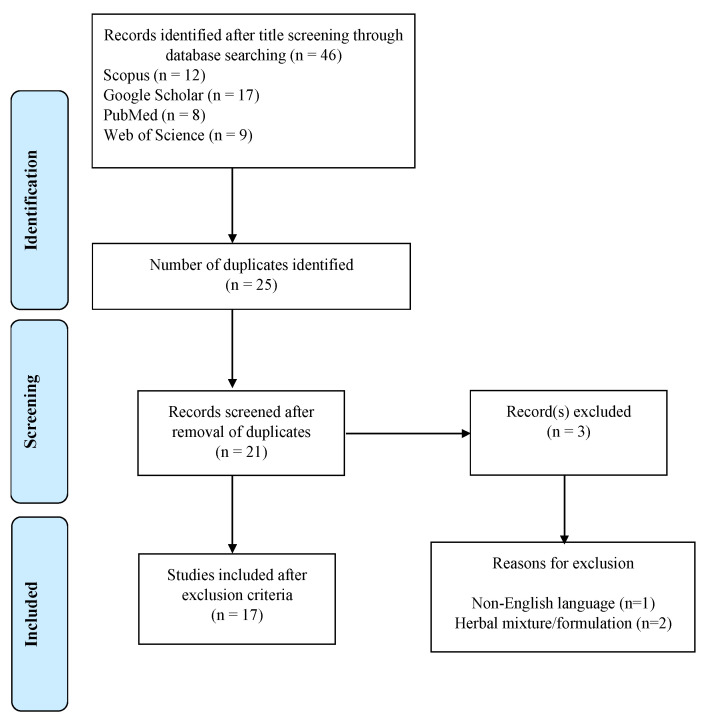
Flow chart used for systematic review.

A total of 46 studies were identified from title screening in different databases (Scopus: *n* = 12; Google Scholar: *n* = 17; PubMed: *n* = 8; and Web of Science: *n* = 9). The number of duplicated articles from these databases was 25 and, after removal of duplicates, 21 articles were obtained. Based on the inclusion and exclusion criteria, 3 studies were excluded (see reasons for exclusion in Figure 1) and 17 articles were included in the study. The studies were published between 2008 and 2021.

## 3. Results and Discussion

### 3.1. General Characteristics

A total of 17 clinical studies were obtained; 16 studies on NS and 1 study on its compound TQ (Table 2). Fourteen studies were randomised while 3 studies [27,28,29] were non-randomised. All were parallel studies. Eight studies were double-blinded and two studies [30,31] were single-blinded. Eleven studies were placebo-controlled, 4 studies used active control: metformin [32,33], received conservative management for diabetic nephropathy [34], atorvastatin + metformin [29], while 2 studies were non-controlled [28,35].

Studies were carried out in Iran (*n* = 7), Egypt (*n* = 2), Saudi Arabia (*n* = 2), Pakistan (*n* = 2), India (*n* = 2), and Indonesia (*n* = 1). All studies were done on adults ≥18 years with the majority up to 60 years with the exception of three studies up to 63 years [36] and 64 years [32,37]. The number of participants analysed ranged from 40 [38] to 99 [31].

All studies enrolled both genders as participants except two studies which did not indicate the gender clearly [28,38]. Ten studies were conducted on TD2 patients, 2 on patients with non-alcoholic fatty liver disease (NAFLD), 1 on diabetic nephropathy patients, 1 on patients with insulin resistance syndrome, 1 on non-diabetic patients with high cholesterol, 1 on patients with metabolic syndrome [31], and 1 on healthy volunteers.

All studies were conducted on the seeds except one study which tested the compound TQ [32]. Only 1 study reported the full information on botanical material including identification of voucher specimen [27]. Most studies tested the oil (*n* = 10) compared to powdered seed (*n* = 6). The majority of studies (*n* = 10) used capsules containing ground seeds or oil, with fewer studies reported the intake of oil directly (*n* = 5) [27,29,34,36,39] and 1 study on tea (hot water extract) [28]. The maximum dose reported were 3 g/day for powdered seed capsule [35], 5 g/day for tea preparation [28], 5 mL/day for oil [36,39], and 100 mg/day for TQ [32]. The duration of the studies varied from 20 days [31], 6 weeks [29,40], 8 weeks [37], 12 weeks [33,41], 6 months [28], to a long-term study of 1 year [30]. Side effect was only reported by 4 studies [32,34,39,41].

**Table 2:** Clinical studies of *Nigella sativa* L. and its compound TQ against diabetes.

Scientific Plant Name (Family)	Part Used/ Compound Tested	Study Design/ Country/ Year	Subjects/Sample Size (after Withdrawal) /Age/Gender	Dosage/ Duration	Continuation of Conventional Therapy during Study	Findings		Side Effects	References
						Treatment (T) Group [Mean Difference]	Control (C) Group/Placebo [Mean Difference]		
*Nigella sativa* L. (Ranunculaceae)	Seed	Randomised double-blind placebo-controlled/Iran/NI	TD2 patients/50 (T: 27, C: 23)/35–64 years/M and F (16/34)	T: 1000 mg NS oil as two capsules, each containing 500 mg NS oil, daily C: two placebo capsules containing medium-chain triglyceride oils in lunch and dinner Duration: 8 weeks	On oral hypoglycemic drug: NI On insulin: No	FBG (mg/dL): 219 ± 64 to 153.6 ± 44.2 [−65.4]	FBG (mg/dL): 172.6 ± 47.2 to 196.4 ± 53.3 [+23.8]	No	[37]
	Seed	Randomised, double-blind, placebo-controlled/ Iran/NI	Patients with NAFLD/43 (T: 22, C: 21)/mean age T: 48.9 ± 12.7, C: 46.2 ± 11.0/M and F (21/22)	T: capsule containing 500 mg-milled edible NS; 2 g/day C: placebo capsules filled by 500 mg rice starch; 2 g/day Duration: 12 weeks	-	FBG (mg/dL): 92.95 ± 15.29 to 85 ± 13.94 [−7.95]	FBG (mg/dL): 95.77 ± 18.29 to 94.55 ± 15.33 [−1.22]	No	[42]
						Fasting insulin (mU/L): 12.32 ± 4.61 to 8.45 ± 4.32 [−3.87]	Insulin (mU/L): 12.46 ± 5.22 to 11.39 ± 6.07 [−1.07]		
						HOMA-IR: 2.82 ± 1.13 to 1.80 ± 1.08 [−1.02]	HOMA-IR: 3 ± 1.44 to 2.72 ± 1.67 [−0.28]		
						QUICKI: 0.332 ± 0.022 to 0.360 ± 0.032 [+0.028]	QUICKI: 0.331 ± 0.025 to 0.337 ± 0.024 [+0.006]		
	Seed	Randomised double-blind placebo-controlled/Iran/NI	TD2 patients/40 (10 in each group)/35–50 years/NI	T1: resistance training + *Nigella sativa* (2 g per day; four capsules containing NS crushed seeds (500 ± 10 mg) per day; two capsules before breakfast and two capsules in the afternoon prior to their food) T2: only *Nigella sativa* T3: resistance training + placebo capsule C: placebo capsule Duration: 8 weeks	On oral hypoglycemic drug: NI On insulin: No	T1 FBG (mg/dL): 142.20 ± 21.11 to 117.20 ± 12.30 [−25] Insulin (µU/mL): 11.02 ± 4.19 to 5.76 ± 2.48 [−5.26] HOMA-S: 71.26 ± 32.35 to 129.05 ± 31.14 [+57.79] HOMA-β: 48.95 ± 7.82 to 45.63 ± 5.90 [−3.32] HOMA-IR: 3.78 ± 1.56 to 1.83 ± 0.71 [−1.95]	FBG (mg/dL): 150.70 ± 19.20 to 142.20 ± 16.94 [−8.5] Insulin (µU/mL): 11.55 ± 2.91 to 10.11 ± 2.75 [−1.44] HOMA-S: 63.72 ± 17.46 to 72.96 ± 20.48 [+9.24] HOMA-β: 46.44 ± 3.23 to 47.47 ± 7.09 [+1.03] HOMA-IR: 4.02 ± 1.09 to 3.51 ± 0.88 [−0.51]	NI	[38]
						T2 FBG (mg/dL): 132.40 ± 23.63 to 129.40 ± 14.81 [−3.0] Insulin (µU/mL): 10.23 ± 3.53 to 9.97 ± 2.25 [−0.26] HOMA-S: 77.18 ± 32.16 to 74.82 ± 20.26 [−2.36] HOMA-β: 58.78 ± 28.50 to 56.83 ± 10.61 [−1.95] HOMA-IR: 3.34 ± 1.39 to 3.24 ± 0.69 [−0.1] T3 FBG (mg/dL): 118.30 ± 17.45 to 119.3 ± 8.43 [+1.0] Insulin (µU/mL): 6.92 ± 2.95 to 7.40 ± 1.37 [+0.48] HOMA-S: 103.81 ± 28.58 to 98.11 ± 24.31 [−5.7] HOMA-β: 53.32 ± 18.82 to 53.09 ± 6.01 [−0.23] HOMA-IR: 2.10 ± 1.24 to 2.23 ± 0.46 [+0.13]			
	Seed	Randomised, double-blind, placebo-controlled/ Iran/2017	Patients with NAFLD/44 (T: 22, C: 22)/20–60 years/M and F (29/15)	T: 1 g of *N.sativa* oil, once a day in capsule C: 1 g of paraffin oil once a day Duration: 8 weeks	-	FBG (mg/dL): 101.13 ± 8.71 to 94.09 ± 7.41 [−7.04]	FBG (mg/dL): 101.40 ± 7.13 to 100.09 ± 7.97 [−1.31]	-	[43]
						Insulin (MU/L): 16.44 ± 5.64 to 17.23 ± 7.55 [+0.79]	Insulin (MU/L): 14.48 ± 3.7 to 14.8 ± 3.59 [+0.32]		
	Seed	Prospective, open-label randomised/Egypt/2016–2018	TD2 patients/44 (T1: 21, T2: 23)/18–60 years/M and F	T1: oil capsules 450 mg three times daily T2: metformin tablets 2000 mg per day Duration: 3 months	On oral hypoglycemic drug: Only group T2 On insulin: no	T1:	T2:	No	[33]
						FBG (mg/dL): 142.5 ± 50.4 to 119.8 ± 23.7 [−22.7]	FBG (mg/dL): 166.2 ± 52.8 to 120.7 ± 25.4 [−45.5]		
	
						2h PPBG (mg/dL): 201.4 ± 48.1 to 184.1 ± 47.5 [−17.3]	2h PPBG (mg/dL): 245.9 ± 60.0 to 171.7 ± 57.9 [−74.2]		
	
						HbA1c (%):7.44 ± 1.16 to 7.01 ± 0.83 [−0.43]	HbA1c (%):7.58 ± 1.63 to 6.55 ± 0.72 [−1.03]		
	
						Insulin sensitivity (%): 59.7 ± 39.0 to 67.3 ± 40.8 [+7.6]	Insulin sensitivity (%): 66.4 ± 32.0 to 83.1 ± 51.4 [+16.7]		
	
						B-cell secretory functions (%): 83.3 ± 52.5 to 97.1 ± 63.7 [+13.8]	B-cell secretory functions (%): 53.2 ± 38.1 to 78.6 ± 47.5 [+25.4]		
	
						Insulin resistance: 2.59 ± 2.00 to 2.20 ± 1.65 [−0.39]	Insulin resistance: 1.87 ± 0.87 to 1.58 ± 0.72 [−0.29]		
	Seed	Randomised, double-blind, placebo-controlled/Iran/2013–2014	TD2 patients/72 (T: 34, C: 33)/30–60 years/M and F	T: 3 g/day oil soft gel capsules (one capsule three times a day) C: sunflower oil as placebo at same dose Duration: 12 weeks	On oral hypoglycemic drug: Yes On insulin: no	FBG (mg/dL): 183.4 ± 42.1 to 166.3 ± 38.5 [−17.1]	FBG (mg/dL): 201.8 ± 63.9 to 204.9 ± 63.2 [+3.1]	One patient in treatment group and two patients in placebo reported stomach ache	[41]
	
						HbA1c (%): 8.3 ± 0.9 to 7.8 ± 0.8 [−0.5]	HbA1c (%): 8.3 ± 1.0 to 8.6 ± 1.0 [+0.3]		
	
						Insulin (mg/dL): 12.2 ± 7.1 to 11.0 ± 3.3 [−1.2]	Insulin (mg/dL): 10.3 ± 9.0 to 13.7 ± 4.6 [+3.4]		
						HOMA-IR: 4.9 ± 2.9 to 4.1 ± 1.3 [−0.8]	HOMA-IR: 4.3 ± 3.9 to 5.7 ± 2.2 [+1.4]		
	Seed	Randomised single-blinded placebo-controlled/Saudi Arabia/2009–2012	TD2 patients on standard oral hypoglycemic drugs/baseline: 103 (T: 51, C: 52), 12 months: 96 (T: 48, C: 48)/18–60 years/M and F	T: powder in capsules of 500 mg; 2 g daily C: activated charcoal capsules (260 mg) as placebo Duration: 1 year	On oral hypoglycemic drug: yes On insulin: no	FBG (mg/dL):	FBG (mg/dL):	No	[30]
						Baseline: 195 ± 6.57	Baseline: 180 ± 5.75		
						3 months: 163.82 ± 6.31 [−31.18]	3 months: 184.90 ± 5.81 [+4.9]		
						6 months: 164.28 ± 5.97 [−30.72]	6 months: 185.80 ± 5.59 [+5.8]		
						9 months: 175.74 ± 6.59 [−19.26]	9 months: 183.88 ± 5.41 [+3.88]		
						12 months: 172.52 ± 5.83 [−22.48]	12 months: 180.25 ± 5.59 [+0.25]		
	
						HbA1c (%):	HbA1c (%):		
						Baseline: 8.6 ± 0.13	Baseline: 8.2 ± 0.12		
						3 months: 7.89 ± 0.18 [−0.71]	3 months: 8.27 ± 0.12 [+0.07]		
						6 months: 7.76 ± 0.22 [−0.84]	6 months: 8.34 ± 0.13 [+0.14]		
						9 months: 7.94 ± 0.19 [−0.66]	9 months: 8.47 ± 0.15 [+0.27]		
						12 months: 8.20 ± 0.14 [−0.4]	12 months: 8.48 ± 0.14 [+0.28]		
	
						C-peptide (ng/mL):	C-peptide (ng/mL):		
						Baseline: 2.9 ± 0.20	Baseline: 2.9 ± 0.20		
						3 months: 2.85 ± 0.18 [−0.05]	3 months: 2.93 ± 0.19 [+0.03]		
						6 months: 2.69 ± 0.17 [−0.21]	6 months: 3.02 ± 0.22 [+0.12]		
						9 months: 2.72 ± 0.19 [−0.18]	9 months: 3.06 ± 0.19 [+0.16]		
						12 months: 2.77 ± 0.17 [−0.13]	12 months: 2.84 ± 0.17 [−0.06]		
	
						Insulin resistance:	Insulin resistance:		
						Baseline: 3.0 ± 0.24	Baseline: 2.5 ± 0.17		
						3 months: 2.52 ± 0.16 [−0.48]	3 months: 2.60 ± 0.16 [+0.1]		
						6 months: 2.42 ± 0.17 [−0.58]	6 months: 2.69 ± 0.19 [+0.19]		
						9 months: 2.48 ± 0.19 [−0.52]	9 months: 2.73 ± 0.16 [+0.23]		
						12 months: 2.50 ± 0.18 [−0.5]	12 months: 2.51 ± 0.15 [+0.01]		
	
						β-cell activity (%):	β-cell activity (%):		
						Baseline: 45.8 ± 3.73	Baseline: 59.4 ± 4.93		
						3 months: 58.65 ± 5.17 [+12.85]	3 months: 57.77 ± 4.09 [−1.63]		
						6 months: 57.63 ± 4.77 [+11.83]	6 months: 58.34 ± 4.34 [−1.06]		
						9 months: 54.86 ± 3.39 [+9.06]	9 months: 59.69 ± 4.15 [+0.29]		
						12 months: 58.57 ± 4.61 [+12.77]	12 months: 56.62 ± 3.51 [−2.78]		
	Seed	Randomised, double-blind, placebo-controlled/Iran/NI	TD2 patients/70/34–63 years/M and F (30/40)	T: 2.5 mL oil daily twice daily taken after the meals C: 2.5 mL mineral oil twice daily taken after the meals Duration: 3 months	On oral hypoglycemic drug: yes On insulin: no	FBG (mg/dL): 180.2 ± 31.8 to 161.9 ± 45.3 [−18.3]	FBG (mg/dL): 179.8 ± 32.3 to 186.3 ± 42.1 [+6.5]	No	[36]
	
						2h PPBG (mg/dL): 183.0 ± 38.7 to 167.9 ± 37.5 [−15.1]	2h PPBG (mg/dL): 189.7 ± 42.8 to 192.2 ± 41.7 [+2.5]		
	
						HbA1c (%): 8.82 ± 0.73 to 8.52 ± 0.68 [−0.3]	HbA1c (%): 8.79 ± 0.55 to 8.70 ± 0.67 [−0.09]		
	Seed	Non-controlled/Egypt/NI	TD2 patients (41) and healthy volunteers (25)/NI	T1: tea (hot water extract) as 5 g/day T2: tea (hot water extract) as 5 g/day in addition to receiving their oral antidiabetic drug Duration: 6 months	On oral hypoglycemic drug: Only group T2 On insulin: NI	T1: Healthy volunteers: FBG (mg/dL): 80.22 ± 10.8 to 73.34 ± 8.71 [−6.88] PPBG (mg/dL): 101.13 ± 15.25 to 89.49 ± 12.38 [−11.64] HbA1c (%): 4.43 ± 0.36 to 4.14 ± 0.47 [−0.29] T2: TD2 patients: FBG (mg/dL): 148.7 ± 26.59 to 127.67 ± 22.01 [−21.03] PPBG (mg/dL): 251.42 ± 76.88 to 164.12 ± 28.72 [−87.3] HbA1c (%): 7.18 ± 0.83 to 6.02 ± 0.58 [−1.16]	-	NI	[28]
	Seed	Randomised, double-blind, placebo-controlled/Iran/NI	Healthy volunteers/70 (T: 35, C: 35)/25–60 years/M and F (35/35)	T: 2.5 mL oil daily two times a day after the meals C: 2.5 mL mineral oil (placebo) two times a day after the meals Duration: 2 months	-	FBG (mg/dL): 102.4 ± 20.8 to 91.5 ± 12.5 [−10.9]	FBG (mg/dL): 98.6 ± 12.0 to 101.0 ± 14.8 [+2.4]	Transient nausea	[39]
	
						HbA1c (%): 5.7 ± 0.7 to 5.3 ± 0.4 [−0.4]	HbA1c (%): 5.6 ± 0.5 to 5.8 ± 0.5 [+0.2]		
	Seed	Randomised non-controlled/Saudi Arabia/NI	TD2 patients/94 (T1: 30, T2: 32, T3: 32)/18–60 years/M and F (43/51)	T1: Capsules of 500 mg grounded seed twice daily (1 g/day) T2: 1 g twice daily (2 g/day) T3: 1 g thrice daily (3 g/day) Duration: 12 weeks	On oral hypoglycemic drug: yes On insulin: NI	T1: FBG (mg/dL): 189 ± 14.3 to 171 ± 7.8 [−18] 2 h PPBG (mg/dL): 286 ± 23.3 to 218 ± 15.6 [−68] HbA1c (%): 8.36 ± 0.31 to 8.01 to ± 0.27 [−0.35] C-peptide (ng/mL): 2.96 ± 0.33 to 3.16 ± 0.32 [+0.2] Insulin resistance index: 2.75 ± 0.34 to 2.82 ± 0.26 [+0.07] Beta cell function (%): 61.75 ± 7.79 to 59.12 ± 8.19 [−2.63] T2: FBG (mg/dL): 219 ± 12.3 to 162 ± 9.2 [−57] 2 h PPBG (mg/dL): 289 ± 24.2 to 256 ± 28.1 [−33] HbA1c (%): 9.09 ± 0.24 to 7.57 ± 0.30 [−1.52] C-peptide (ng/mL): 3.02 ± 0.32 to 2.66 ± 0.26 [−0.36] Insulin resistance index: 3.20 ± 0.36 to 2.37 ± 0.20 [−0.83] Beta cell function (%): 45.03 ± 6.28 to 63.63 ± 9.59 [+18.6] T3: FBG (mg/dL): 204 ± 18.2 to 169 ± 16.4 [−35] 2 h PPBG (mg/dL): 277 ± 54.3 to 234 ± 80.3 [−43] HbA1c (%): 9.35 ± 0.41 to 7.31 ± 0.37 [−2.04] C-peptide (ng/mL): 3.54 ± 0.36 to 3.44 ± 0.47 [−0.1] Insulin resistance index: 4.11 ± 0.55 to 2.98 ± 0.49 [−1.13] Beta cell function (%): 41.89 ± 9.83 to 88.90 ± 36.05 [+47.01]	-	No	[35]
	Seed	Placebo-controlled/Pakistan/NI	TD2 patients/41/30–60 years/M and F	T: oil (obtained from 0.7 g seeds) for 40 days followed by a placebo (wheat bran) for another 40 days Duration: 80 days	On oral hypoglycemic drug: yes On insulin: no	FBG (mg/dL): 190.78 ± 8.04 to 168.32 ± 7.15 [−22.46]	FBG (mg/dL): 168.32 ± 7.15 to 186.49 ± 7.49 [+18.17]	No	[27]
	
						Insulin (ulU/mL): 8.01 ± 0.76 to 13.19 ± 1.40 [+5.18]	Insulin (ulU/mL): 13.19 ± 1.40 to 8.85 ± 0.69 [−4.34]		
	Seed	Prospective, randomised, parallel group, and open-label/India/2014–2015	Diabetic nephropathy patients/63 (T: 32, C: 31)/20–60 years/M and F	T: oil (2.5 mL once daily) along with conservative management C: received conservative management ((insulin, torsemide, telmisartan, iron, calcium, Vitamin D3, and erythropoietin) Duration: 12 weeks	Received conservative management of diabetic nephropathy	FBG (mg/dL): 138.14 ± 33.13 to 104.09 ± 9.30 [−34.05]	FBG (mg/dL): 114.27 ± 22.00 to 103.82 ± 13.18 [−10.45]	Nausea, diarrhea, rashes, altered taste, the reactions were mild	[34]
	
						PPBG (mg/dL): 190.50 ± 66.13 to 143.14 ± 15.93 [−47.36]	PPBG (mg/dL): 163.91 ± 32.07 to 141.64 ± 15.09 [−22.27]		
	Seed	Prospective study/India/2006–2007	Patients of insulin resistance syndrome/60/M and F (50/10)	T1: atorvastatin 10 mg once a day, tablet metformin 500 mg twice a day, and NS oil 2.5 mL twice daily T2: atorvastatin 10 mg once a day and tablet metformin 500 mg twice a day Duration: 6 weeks	On oral hypoglycemic drug: yes On insulin: NI	T1: Reduction in FBG (mg/dL): [−29.24 ± 6.09] Reduction in PPBG (mg/dL): [−23.39 ± 8.54] T2: Reduction in FBG (mg/dL): [−18.46 ± 6.77] Reduction in PPBG (mg/dL): [−19.87 ± 6.22]	-	NI	[29]
	Seed	Randomised single-blinded placebo-controlled/Indonesia/2016	Patients with metabolic syndrome/99 (T1: 33, T2: 33, C: 33)/>18 years/M and F (23/76)	T1: 1.5 mL/day of oil in capsule T2: 3 mL/day of oil in capsule C: placebo Duration: 20 days	On oral hypoglycemic drug: yes On insulin: NI	T1: HbA1c (%): 8.56 ± 2.78 to 7.44 ± 2.49 [−1.12] T2: HbA1c (%): 9.34 ± 3.30 to 7.51 ± 2.49 [−1.83]	HbA1c (%): 9.30 ± 8.06 to 9.60 ± 2.26 [+0.3]	NI	[31]
	Seed	Randomised, double-blind placebo-controlled/Pakistan/2006–2007	Non-diabetic patients with high cholesterol/ 73 (T: 39, C: 34)/≥ 18 years/M and F	T: two capsules of 500 mg twice daily after meals (1 g twice daily) C: placebo capsules (calcium lactate powder) Duration: 6 weeks	-	FBG (mg/dL): 95.76 ± 16.79 to 86.01 ± 18.36 [−9.75]	FBG (mg/dL): 98.37 ± 12.37 to 90.27 ± 23.78 [−8.1]	No	[40]
	Thymoquinone	Randomised, open label, prospective, three-arm, parallel, multicenter/NI/NI	TD2 patients/45 (T1: 13, T2: 18, C: 14)/27–64 years/M and F (32/28)	T1: 1 tablet of metformin SR 1000 mg once daily and 1 tablet of TQ 50mg T2: 1 tablet of metformin SR 1000 mg and 2 tablets of TQ 50 mg daily C: Active control: 1 tablet of metformin SR 1000 mg Duration: 90 days	On oral hypoglycemic drug: yes On insulin: NI	T1: FBG (mg/dL): 144.0 ± 21.6 to 114.3 ± 8.6 [−29.7] PPBG (mg/dL): 199.8 ± 28.5 to 147.9 ± 10.7 [−51.9] HbA1c (%): 7.2 to 6.7 [−0.5] T2: FBG (mg/dL): 118.0 ± 5.6 to 103.2 ± 4.4 [−14.8] PPBG (mg/dL): 180.3 ± 12.4 to 138.6 ± 6.6 [−41.7] HbA1c (%): 7.2 to 6.8 [−0.4]	FBG (mg/dL): 129.7 ± 6.9 to 111.4 ± 6.3 [−18.3] PPBG (mg/dL): 191.1 ± 10.8 to 161.7 ± 7.7 [−29.4] HbA1c (%): 7.3 to 7.1 [−0.2]	Diarrhea, epigastric pain, abdominal pain and stomachache	[32]

NI: not indicated; C: control group; T: treatment group; FBG: fasting blood glucose; HbA1c: hemoglobin A1C; PPBG: postprandial blood glucose; HOMA-IR: homeostatic model assessment for insulin resistance; HOMA-β: homeostatic model assessment for assessment of beta-cell functionality; HOMA-S: homeostatic model assessment for insulin sensitivity; QUICKI: quantitative insulin-sensitivity check index.

### 3.2. Quality Assessment

Out of the 17 clinical studies, the Jadad quality assessment was carried out on the 13 randomised controlled studies. Eleven studies had high methodological quality; only one study obtained a perfect score of 5 [41]. Although all 13 randomised controlled studies indicated randomisation in their methodology, only 6 mentioned how the randomisation was conducted, which included computer (software) generated random numbers [32,33,34,41], table of random numbers [30], and lottery system [31]. Three studies [38,42,43] did not indicate any method of randomisation. Four studies [36,37,39,40] reported the use of block randomisation but did not indicate how the generation of sequence of randomisation was conducted to know if each study participant had the same chance of receiving each intervention. 

Furthermore, among the 13 randomised controlled studies, 8 studies were described as double-blinded, of which 6 indicated an appropriate method of blinding using the following statements: “the researchers and participants remained blind”, also, “supplements (placebo and NS) had similar appearance” [37], “the researchers and participants remained blind”, also, “NS and sunflower soft gel capsules were prepared…in the same size and color” [41], “Supplement boxes were labeled as A or B to blind the investigators and patients to group assignments. Labeling was done by a third person” [42], “both the patient as well as the investigators were unaware of the treatment group they were assigned, capsules in experimental and placebo groups were identical”, also, “one could not guess the capsule contents by looking at the blister packs” [40], “Capsules in experimental and placebo groups were identical in appearance” [38], “the appearance and flavor of mineral oil similar to NS oil”, also, “The NS and mineral oils were filled separately into 150 mL bottles and labeled as A and B” [36]. 

In addition, 9 out of the 13 randomised controlled studies provided a proper description for withdrawals and dropouts, among which three studies [36,38,39] stated that no withdrawal occurred, and all participants completed the intervention. The details of the quality assessment of individual studies are provided in Table 3.

### 3.3. Findings of Systematic Review 

Kooshki et al. [37] investigated the effect of 1 g/day of NS oil for 8 weeks in 50 TD2 patients (NS: 27, placebo: 23). FBG was decreased significantly (*p* < 0.001) in the NS oil group compared with baseline [219 ± 64 to 153.6 ± 44.2 mg/dL (−65.4)], whereas no significant change was observed in the placebo group [172.6 ± 47.2 to 196.4 ± 53.3 mg/dL (+23.8)]. Another study by Darand et al. [42] evaluated the effects of lifestyle modification plus 2 g/day powder of either NS or placebo for 12 weeks in 43 patients with NAFLD (NS: 22, placebo: 21). Compared with placebo, NS significantly reduced the level of glucose (−7.95 vs.−1.22 mg/dL; *p* = 0.041), insulin (−3.87 vs. −1.07 mU/L; *p* = 0.027), HOMA-IR (−1.02 vs. −0.28; *p* = 0.021), and significantly increased QUICKI (0.03 vs. 0.006; *p* = 0.002). Moreover, Jangjo-Borazjani et al. [38] studied the effects of NS during resistance training for 8 weeks in 40 TD2 patients (10 patients in each of the 4 groups; resistance training + NS, NS alone, resistance training + placebo capsule, and placebo capsule alone). Resistance training or NS (2 g/day seed powder) individual treatments reduced HOMA-IR, insulin, glucose, and increased HOMA-S. The combination of both treatments, rather than each intervention alone, had significant effects on the reduction in HOMA-IR and insulin, as well as increased HOMA-β/S. Comparison of NS treatment alone and the placebo group showed no clear differences.

Furthermore, Moustafa et al. [33] found that 1.35 g/day NS for 3 months in newly diagnosed TD2 patients (NS: 21 patients, metformin: 23 patients) was inferior to metformin (2 g/day) in lowering FBG (−22.7 vs. −45.5 mg/dL), 2 h PPBG (−17.3 vs. −74.2 mg/dL), and HbA1c (−0.43 vs. −1.03%) but was comparable to metformin in terms of their effects on fasting insulin (−1.7 vs. −1.2), insulin sensitivity (+7.6 vs. +16.7%), and insulin resistance (−0.39 vs. −0.29). Heshmati et al. [41] investigated the effect of 3 g/day oil soft gel capsules for 12 weeks in 72 TD2 patients (NS: 34, placebo: 33). FBG (−9.1%) and HbA1c (−5.1%) changed significantly in the treatment group compared to baseline (*p* < 0.05). Moreover, insulin level and insulin resistance were reduced in the treatment group, but after adjusting for weight changes, dietary changes and baseline values, the changes were not significant. Comparison of NS and placebo showed clear differences in FBG (−17.1 vs. +3.1 mg/dL), HbA1c (−0.5 vs. +0.3%), insulin (−1.2 vs. +3.4 mg/dL), and HOMA-IR (−0.8 vs. +1.4).

In addition, Kaatabi et al. [30] evaluated the effect of 2 g/day of NS, in addition to standard medications, for one year in 103 analysed TD2 patients (NS: 51, placebo: 52). Compared to placebo, a significant drop was observed in FBG in NS (−31.18 vs. +4.9 mg/dL at 3 months and −22.48 vs. +0.25 mg/dL at 12 months) and HbA1c (−0.84 vs. +0.14% at 6 months and −0.4 vs. +0.28% at 12 months). Similarly, differences were observed in C-peptide level (−0.21 vs. +0.12 ng/mL at 6 months and −0.13 vs. −0.06 ng/mL at 12 months), insulin resistance (−0.58 vs. +0.19 at 6 months) and β-cell activity (+12.85 vs. −1.63 at 3 months and +12.77 vs. −2.78 at 12 months).

Moreover, Bilal et al. [27] studied the effect of NS oil in TD2 patients. The mean glucose level was reduced from 190.780 ± 8.042 to 168.317 ± 7.150 mg/dL (−22.46) after NS treatment for 40 days and again increased to 186.487 ± 7.491 mg/dL [+18.17] after placebo administration for a similar period. The mean insulin level increased from 8.013 ± 0.758 ulU/mL to 13.194 ± 1.404 ulU/mL [+5.18] after NS treatment and again reduced to 8.850 ± 0.694 ulU/mL [−4.34] after placebo intervention. 

Rashidmayvan et al. [43] studied the effect of NS oil (1 g/day) for 8 weeks on serum levels of inflammatory markers, liver enzymes, lipid profile, insulin, and FBG in 44 patients with NALFD (22 patients in each group). Differences were observed in FBG in the NS group compared to placebo (−7.04 vs. −1.31 mg/dL). No significant effect was observed on serum levels of insulin.

Hosseini et al. [36] explored the possible anti-hyperglycemic effect of NS oil (5 mL/day) in 70 TD2 patients. A significant decrease was observed in the NS oil group compared to the placebo group with regards to FBG (−18.3 vs. +6.5 mg/dL), 2h PPBG (−15.1 vs. +2.5 mg/dL), and HbA1c levels (−0.3 vs. −0.09%).

In the study of El-Shamy et al. [28] on the effect of NS tea (hot water extract as 5 g/day for 6 months) on 41 TD2 patients and 25 healthy volunteers, a reduction was observed in FBG, PPBG, and HbA1c, which was greater among TD2 patients compared to healthy individuals (FBG: −21.03 vs. −6.88 mg/dL; PPBG: −87.3 vs. −11.64 mg/dL; HbA1c: −1.16 vs. −0.29%].

Mohtashami et al. [39] explored the effect of NS oil (5 mL/day for 2 months) on 70 healthy subjects (35 subjects each in treatment and placebo group). A significant decrease in FBG and HbA1c levels in NS-treated patients was observed as compared to the control group (FBG: −10.9 vs. +2.4 mg/dL and HbA1c: −0.4 vs. +0.2%).

Bamosa et al. [35] evaluated 3 doses (1 g, 2 g, and 3 g/day) of NS seed powder for 12 weeks in 96 TD2 patients (divided into three groups of 30, 32, and 32 patients for the 3 doses, respectively). The dose 3 g/day was more effective compared to 2 g/day in improving all parameters; PPBG (−43 vs. −33 mg/dL), HbA1c (−2.04 vs. −1.52%), insulin resistance (−1.13 vs. −0.83), and beta cell function (+47.01 vs. +18.6%), with the exception of FBG (−35 vs. −57 mg/dL) and C-peptide (−0.1 vs.−0.36).

Ansari et al. [34] assessed the protective role of NS (2.5 mL/day for 12 weeks) in 63 patients with diabetic nephropathy (NS: 32, control: 31). NS showed a significant drop in FBG (−34.05 vs. −10.45 mg/dL) and PPBG (−47.36 vs. −22.27 mg/dL) compared to control group.

Najmi et al. [29] studied the effect of NS oil (5 mL/day for 6 weeks) on various clinical and biochemical parameters in 60 patients with insulin resistance syndrome. Patients were divided into two groups of 30 each; Group 1: atorvastatin 10 mg once a day and tablet metformin 500 mg twice a day and Group 2: atorvastatin 10 mg once a day, tablet metformin 500 mg twice a day, and NS oil 2.5 mL twice daily. The NS group showed a slightly greater reduction in both FBG (−29.24 ± 6.09 vs. −18.46 ± 6.77 mg/dL) and PPBG (−23.39 ± 8.54 vs. −19.87 ± 6.22 mg/dL) compared to the other group.

Rachman et al. [31] studied the efficacy of NS oil (1.5 mL and 3 mL/day) and hypoglycemic drug combination for 20 days to reduce HbA1c levels in patients with metabolic syndrome risk. Ninety-nine patients were divided into three groups of 33 each. Reduction in HbA1c was greater in the 3 mL/day group compared to 1.5 mL/day (−1.83 vs. −1.12%) while placebo displayed a difference of +0.3%.

In a study by Qidwai et al. [40] on NS seed powder (2 g/day for 6 weeks) in 73 non-diabetic patients (treatment:39, placebo:34), no significant difference was observed in FBG between NS and placebo (−9.75 vs. −8.1 mg/dL).

A 90-day randomised, open-label, prospective, three-arm, parallel, multicentre study was carried out by Ali et al. [32] to assess the safety and efficacy of TQ administration with metformin in TD2 patients divided into 3 groups. Group 1 (T1) received 1 tablet of metformin SR 1000 mg + 1 tablet of TQ 50mg once daily. Group 2 (T2) was given 1 tablet of metformin SR 1000 mg + 2 tablets of TQ 50mg once daily. Group 3 (R) obtained 1 tablet of metformin SR 1000 mg only. The HbA1c values in T1, T2 and R decreased from 7.2, 7.2 and 7.3 to 6.7, 6.8, and 7.1, respectively, after 3 months. A significant number of patients displayed decreased HbA1c < 7% as follows; T1: 69.2% patients, T2: 61.1% patients and R: 21.4% patients at 90 days. A greater reduction in FBG and PPBG was also observed in T1 [FBG from 144.0 ± 21.6 to 114.3 ± 8.6 mg/dL (−29.7); PPBG from 199.8 ± 28.5 to 147.9 ± 10.7 mg/dL (−51.9)] and T2 [FBG from 118.0 ± 5.6 to 103.2 ± 4.4 mg/dL (−14.8); PPBG from 180.3 ± 12.4 to 138.6 ± 6.6 mg/dL (−41.7)] compared to group 3 [FBG from 129.7 ± 6.9 to 111.4 ± 6.3 mg/dL (−18.3); PPBG from 191.1 ± 10.8 to 161.7 ± 7.7 mg/dL (−29.4)].

The detailed findings of all studies on NS and TQ are presented in Table 2.

### 3.4. Safety Assessment

In the randomised, double-blind, placebo-controlled study by Heshmati et al. [41] on *Nigella sativa* L. oil in TD2 patients, one patient in the treatment group and two patients in placebo reported stomachache. In another study by Mohtashami et al. [39], no adverse reaction was noted by any volunteers except transient nausea in the NS oil-treated group. Moreover, in the study of Ansari et al. [34], the signs and symptoms at the beginning of the treatment were similar in NS and control groups, comprising anorexia, nausea, vomiting, weakness, weight loss, pruritus, edema, oliguria, and anemia. These symptoms eventually improved in both groups after 12 weeks of treatment, but this was more apparent in the NS oil-treated group.

In addition, in the randomised, open-label, prospective study by Ali et al. [32] on the clinical efficacy of TQ, a total of 13 adverse events were reported by 11 (18.3%) of the 60 patients in the study. In group T1 (receiving 1 tablet of metformin SR 1000 mg and 1 tablet of TQ 50 mg once daily), 3 (15%) patients reported 4 adverse effects. Seven adverse events were noted by 6 (28.6%) patients in group T2 (receiving 1 tablet of metformin SR 1000 mg + 2 tablets of TQ 50 mg once daily), while 2 (10.5%) subjects in group R (1 tablet of metformin SR 1000 mg only) experienced 2 adverse events. The adverse events reported were diarrhoea, epigastric pain, abdominal pain, and stomachache. The outcome of the adverse effect was “resolved” for all 13 adverse events and none were reported as serious (Table 2).

### 3.5. Mechanism of Action of NS and TQ

Studies have proved several mechanisms of action of the antidiabetic properties of NS. Dalli et al. [44] evaluated the chemical composition of different NS fractions using GC-MS for the esterified fatty acids or HPLC-UV for the organic fraction as well as their in vitro/in vivo inhibitory effect on pancreatic α-amylase and intestinal glucose absorption. The n-hexane fraction was characterised by the presence of linoleic acid (44.65%), palmitic acid (16.32%), stearic acid (14.60%), and TQ (8.7%). In the ethanolic fraction, catechin (89.03 mg/100 g dry weight (DW)), rutin (6.46 mg/100 g DW), and kaempferol (0.032 mg/100 g DW) were found. The methanolic fraction was marked by the existence of gallic acid (19.91 mg/100 g DW), catechin (13.79 mg/100 g DW), and rutin (21.07 mg/100 g DW), while the aqueous fraction was distinguished with the presence of salicylic acid (32.26 mg/100 g DW), rutin (21.46 mg/100 g DW), and vanillic acid (3.81 mg/100 g DW). With regard to the in vitro inhibitory effect on pancreatic α-amylase, ethanol fraction displayed IC_50_ of 0.25 mg/mL, methanol (IC_50_ = 0.10 mg/mL), aqueous (IC_50_ = 0.31 mg/mL), and n-hexane fraction (IC_50_ = 0.76 mg/mL). α-amylase inhibition was also observed in normal and diabetic rats. Moreover, the percentage of intestinal glucose absorption for all tested extracts ranged from 24.82 to 60.12%.

Tiji et al. [45] studied the in vitro inhibitory effect of NS extracts and fractions against intestinal α-glucosidase and pancreatic α-amylase. The acetone fraction SA3 exhibited a high inhibitory effect (72.26 ± 1.42%) on intestinal α-glucosidase activity, comparable to acarbose (70.90 ± 1.12%). The acetone fractions also showed an inhibitory effect close to that of acarbose against pancreatic α-amylase. Specifically, the SA2 fraction displayed an inhibitory effect of 67.70 ± 0.58% and showed a high amount of apigenin and gallic acid. The compounds apigenin, (−)-catechin, and gallic acid were further characterised for their enzyme inhibitory properties. Interestingly, (−)-catechin displayed a possible synergistic enzyme inhibitory effect with acarbose against α-glucosidase, while apigenin showed an additive inhibitory effect with acarbose against α-amylase.

Fararh et al. [46] found that the hypoglycemic effect of NS oil is partly due to a decrease in hepatic gluconeogenesis. Glucose production was significantly reduced in hepatocytes isolated from NS oil-treated hamsters after 2 h incubation with gluconeogenic precursors [alanine, glycerol and lactate], in comparison to hepatocytes isolated from untreated diabetic animals. The study also showed that TQ reduces hepatic glucose production in diabetic hamsters. In another study by Kanter et al. [47], NS displayed a therapeutic protective effect in STZ-induced diabetic rats by decreasing oxidative stress and preserving pancreatic β-cell integrity. Moreover, Kanter et al. [48] investigated the protective effects of the volatile oil of NS seeds on insulin immunoreactivity and ultrastructural changes of pancreatic β-cells in STZ-induced diabetic rats. NS exhibited a therapeutic protective effect by decreasing morphological changes and preserving pancreatic β-cell integrity

Furthermore, Dong et al. [49] studied the effect of NS seed polysaccharides on TD2 mice and gut microbiota. NS significantly increased the number of *Muribaculaceae* Unclassified and *Bacteroides*, which were significantly suppressed in mice gut after STZ treatment. Additionally, treatment of rats with NS extract and oil, as well as the compound TQ, significantly reduced diabetes-induced increases in pancreatic tissue malondialdehyde and serum glucose and significantly raised serum insulin and tissue superoxide dismutase. Ultrastructurally, TQ ameliorated the adverse effects of STZ, such as segregated nucleoli, heterochromatin aggregates, and mitochondrial vacuolisation and fragmentation [50].

Moreover, Ali et al. [51] found that NS mediated its antidiabetic effects via activation of insulin and AMP-activated protein kinase (AMPK) pathways, and by mitochondrial uncoupling. NS was found to activate AKT and ERK1/2 in C2C12 myotubes to values nearly 50% greater than the vehicle dimethyl sulfoxide. Similarly, NS increased the phosphorylation of AMPK and ACC in a similar manner to the positive control AICAR. In H4IIE hepatocytes, NS increased the phosphorylation of AKT, decreased ERK1/2 activation, and stimulated the hepatic AMPK pathway. On the contrary, NS showed no effect on either the insulin or the AMPK pathway in 3 T3-L1 adipocytes. Finally, NS dose-dependently (25 to 200 μg/mL) lowered the oxygen consumption of isolated liver mitochondria. Moreover, Balbaa et al. [52] observed that administration of NS oil significantly induced the gene expression of insulin receptor and upregulated the expression of insulin-like growth factor-1 and phosphoinositide-3 kinase, while the expression of ADAM-17 was downregulated. 

With regard to the compound TQ, oral administration for 45 days dose-dependently improved the glycemic status in STZ–nicotinamide-induced diabetic rats by increasing the levels of insulin, decreasing glucose and HbA1C levels, and restoring the altered activities of carbohydrate metabolic enzymes to near normal. In addition, reduced activities of hexokinase, glucose 6-phosphate dehydrogenase and increased activities of gluconeogenic enzymes glucose 6-phosphatase and fructose 1, 6-bisphosphatase were seen in diabetic rats. TQ significantly reversed the activities of these enzymes to near normal [53].

El-Mahmoudy et al. [54] showed the protective effect of TQ against Type 1 diabetes development via the nitric oxide inhibitory pathway. Nitric oxide is involved in β-cell destruction during the development of Type 1 diabetes mellitus. TQ showed no effect on either I*k*B degradation or NF-*k*B activation; nonetheless, it significantly inhibited both p44/42 and p38 mitogen-activated protein kinases (MAPKs) which contribute to the transcriptional machinery of inducible nitric oxide synthase and nitric oxide production. Rani et al. [55] also observed that TQ nanocapsules (containing half of the doses of TQ) produced a better antihyperglycemic effect in comparison to TQ alone in a TD2 rat model. In another study by Abdelrazek et al. [56], both pancreatic and hepatic catalase and glutathione activities showed a significant increment in diabetic rats treated with NS oil. NS oil also improved the histopathological picture and hepatic glycogen contents in the diabetic rats and increased insulin immunoreactive parts % and the mean pancreatic islet diameter. Moreover, oral administration of TQ at 80 mg/kg b.w. in diabetic rats for 45 days significantly improved the glycoprotein changes (levels of hexose, hexosamine, fucose, and sialic acid) [57].

On top of that, NS aqueous extract and TQ significantly suppressed the expression of COX-2 enzyme in the pancreatic tissue of STZ diabetic rats. NS and TQ treatment also suppressed pancreatic tissue lipid peroxidation malondialdehyde levels and raised the level of superoxide dismutase antioxidant enzyme correlated with the decrease in COX-2 mRNA expression [58]. In addition, Ahmad et al. [59] observed that TQ exerted a synergistic effect with glibenclamide on glucose levels in rats. The maximum reduction in blood glucose level (47.4%) was observed 3 h following glibenclamide administration, while co-administration with TQ caused a reduction of 53.0% to 56.2%. Moreover, co-administration of TQ as single and multiple doses raised the plasma concentration of glibenclamide by 13.4% and 21.8%, respectively. The area under plasma concentration-time curve (AUC) and half-life (T1/2) of glibenclamide were also increased by 32.0% and 17.4%, respectively, with a TQ single dose, and by 52.5% and 92.8%, respectively, after chronic treatment. Moreover, TQ resulted in a marked decrease in hepatic protein expressions of CYP3A2 and CYP2C11 enzymes which are responsible for the metabolism of glibenclamide.

Furthermore, in the in silico study carried out by Megantara et al. [60], TQ acts as a peroxisome proliferator-activated receptor gamma (PPAR-γ) agonist in the treatment of TD2. TQ interacts in the binding pocket 1 of the B chain and binding pocket 2 of the A chain in the same interaction with pioglitazone. Despite the fact that the binding affinity of TQ was found to be lower than pioglitazone to PPAR-γ, [binding affinity and inhibition constant values Ei = −9.4 kcal/mol; Ki = 0.13 µM (pioglitazone) and Ei = −7.0 kcal/mol; Ki = 7.43 µM (TQ)], TQ can potentially be developed as a PPAR-γ agonist compound.

Moreover, TQ significantly improved insulin sensitivity in diabetic rats, which was confirmed by an increased level of PPAR-γ and reduced HOMA-IR. Molecular docking of TQ displayed a substantial binding affinity with PPAR-γ and DPP-IV target proteins. The docked TQ interacted with PPAR-γ with a binding energy of −5.7 kcal/mol, while it showed better binding affinity with DPP-IV targets (−6.3 kcal/mol). The most stable conformation of TQ in the ligand binding site of PPAR-γ is encircled by amino acid residues including Ile262, Lys263, Gly284, Cys285, Arg288, Leu330, Leu333, Leu340, Ile341, and Ser342. As for DPP-IV, the inhibitor binding cavity was utilised by TQ and surrounded by the amino acid residues Tyr547, Ser630, Tyr631, Val656, Trp659, Tyr662, Tyr666, and Val711. In addition, docked TQ upon superposition with a known inhibitor of DPP-IV displayed two robust hydrogen bond interactions with Tyr547 and Tyr662 residues [61].

### 3.6. Pharmacokinetics

Thymoquinone has been reported to exhibit slow absorption and rapid elimination properties when administered perorally. Recently, novel drug delivery systems especially nanoparticulate drug delivery systems have gained great interest due to their enhancement of the pharmacokinetics such as absorption, distribution, metabolism, and excretion of drug. Moreover, these nanoformulations possess key advantages over conventional formulations in several ways including (i) enhancement of solubility and bioavailability, (ii) targeted drug delivery, (iii) sustained drug release, (iv) reduced dosage amount, and (v) reducing possible side effects [55].

A study by Alkharfy et al. [62] evaluated the pharmacokinetic profile of TQ following intravenous (IV) and oral (PO) administration in rabbits. The mean plasma concentration–time curve showed a rapid poly-exponential decline, and TQ displayed linear kinetics at a dose of 5 mg/kg via IV administration. The calculated absolute bioavailability of TQ was ~58% with a lag time of ~23 min while the estimated TQ protein binding was >99%. Therefore, TQ displayed rapid elimination and relatively slower absorption after PO administration, but with good bioavailability.

In addition, Iqbal et al. [63] investigated the pharmacokinetic behaviour of TQ following PO and IV administration in layer chickens. Maximum plasma concentration (C_max_) following PO and IV administration was 8.805 and 4.497 µg/mL, respectively, while the time to reach maximum concentration (T_max_) was 1 and 0.1 h, respectively. Moreover, the elimination half-lives were 1.02 and 0.978 h, while the mean residence times were 1.79 and 1.036 h respectively. The absolute bioavailability was found to be almost 85%.

Kumar et al. [64] carried out an in vitro study to evaluate the stability and bioavailability of TQ encapsulated in the developed nanocarrier. The percentage micelleration of TQ from the developed nanocapsules was greater in comparison to that of mixed micelles. Moreover, the everted gut sac methodology resulted in higher absorption of nanoencapsulated products in comparison to mixed micelles, confirming an improvement in bioavailability of TQ via formulated nanocapsules.

In another study by Ahmad et al. [65], the gender-dependent pharmacokinetic behaviour of TQ was studied in rats following the administration PO (20 mg/kg) and IV (5 mg/kg). Gender difference did not seem to have a significant role in TQ disposition at steady state. Following PO administration, the C_max_ of TQ was 4.52 ± 0.092 μg/mL in male rats and 5.22 ± 0.154 μg/mL in female rats while after IV administration, the C_max_ was 8.36 ± 0.132 μg/mL and 9.51 ± 0.158 μg/mL, respectively. Moreover, the area under the plasma concentration-time curve (AUC)_0–__∞_ following PO administration was 47.38 ± 0.821 μg/mL h in females and 43.63 ± 0.953 μg/mL h in males.

Furthermore, the pharmacokinetic profile of TQ-loaded nanostructured lipid carriers was evaluated in rabbits [66]. The pharmacokinetic properties of TQ were improved. The time needed to reach maximum concentration (T_max_), C_max_, and elimination half-life of TQ were found to be 3.96 ± 0.19 h, 4811.33 ± 55.52 ng/mL, and 4.4933 ± 0.015 h, respectively, showing that TQ is suitable for extravascular administration. Another study by Ansar et al. [67] also determined the bioavailability of TQ-loaded nanostructured lipid carriers following PO and IV administration in rats. The complex displayed better absorption when administered IV compared to PO administration. However, PO administration had greater bioavailability in comparison to the IV route.

Additionally, a cationic liposomal formulation of TQ displayed significantly higher in vivo absorption, approximately 1.5-fold higher plasma concentration, greater bioavailability, decreased volume of distribution and improved clearance relative to TQ [68]. Moreover, a self-nanoemulsifying drug delivery system was designed using the microemulsifcation technique. Zeta potential was found to be −11.35 mV, indicating the high stability of the oil droplets. Pharmacokinetic assessment in rats revealed a fourfold increase in the bioavailability of the TQ-self-nanoemulsifying drug delivery system over pure TQ [69]. Similarly, the relative bioavailability of TQ was enhanced 3.87-fold by a self-nanoemulsifying drug delivery system in comparison with TQ suspension [70].

Moreover, Rahat et al. [71] developed chitosan-modified solid lipid nanoparticles to improve the oral bioavailability of TQ. The nanoparticle complex displayed particle size, polydispersity index, and drug entrapment in the range between 135.61 and 211.36 nm, 0.17–0.29, and 65.14–91.78% and zeta potential of +12.52 ± 1.21 mV. Moreover, the TQ-chitosan-modified solid lipid nanoparticles showed a controlled release profile during 24 h of study and also displayed excellent mucoadhesion with 67.26 ± 2.18 % mucoadhesive efficiency. Absorption of TQ was much higher and faster following the TQ-nanoparticle complex administration, displaying AUC_0→24_ value and C_max_ of 1713.88 μ∙h/mL and 169.73 μg/mL, respectively. A 3.53-fold improvement in relative oral bioavailability was observed compared to pure TQ suspension. The T_max_, MRT, t_1/2_, and K_el_ of TQ-chitosan-modified solid lipid nanoparticles were found to be 2 h, 8.22 h, 10.81 h, and 0.064 h^−1^, respectively.

### 3.7. Overall Discussion

From the findings of the clinical studies, NS was found to be highly potent in terms of its hypoglycemic activity when compared to placebo. For instance, NS displayed high hypoglycemic effect in TD2 patients with a mean difference in FBG of −65.4 mg/dL [37], −57 mg/dL [35], −31.18 mg/dL [30], −22.7 mg/dL [33], −22.46 mg/dL [27], −21.03 mg/dL [28], −18.3 mg/dL [36], and −17.1 mg/dL [41]. The FBG reduction was lower in patients with NALFD; −7.95 mg/dL [42] and −7.04 mg/dL [43], and also lower in healthy participants; −10.9 mg/dL [39], −9.75 mg/dL [40], and −6.88 mg/dL [28]. PPBG was also reduced significantly by −87.3 mg/dL [28], −47.36 mg/dL [34], −43 mg/dL [35], −17.3 mg/dL [33], and −15.1 mg/dL [36]. HbA1c was reduced by −2.04% [35], −1.83% [31], −1.16% [28], −0.84% [30], −0.5% [41], and −0.3% [36]. Moreover, HOMA-IR was reduced by −1.13 [35], −1.02 [42], −0.8 [41], and −0.58 [30] while β-cell activity was increased by +47.01% [35] and +12.85% [30].

Few meta-analyses have been previously conducted on NS. For instance, seven studies were included in the meta-analysis of Daryabeygi-Khotbehsara et al. [6]. NS significantly improved FBG (−17.84 mg/dL, 95% CI: −21.19 to −14.49) and HbA1c (−0.71%, 95% CI: −1.04 to −0.39). No evidence of heterogeneity was observed (I^2^ = 28.5%) for FBG. Moreover, a subgroup analysis assessed the efficacy of supplementation form (powder or oil) on FBG which was significantly lowered in both studies which used powder supplement [weighted mean difference (WMD): −18.0 mg/dL, 95% CI: −20.1 to −15.9] and oil supplement (WMD: −20.7 mg/dL, 95% CI: −27.0 to −14.5). With regard to HbA1c, significant evidence of heterogeneity across studies was noted (I^2^ = 89.3%). HbA1c level was lowered with both forms of NS (oil: WMD= −0.46%, 95% CI: −0.65 to −0.28, powder: WMD= −0.98%, 95% CI: −1.05 to −0.92). Heterogeneity was demonstrated across studies evaluating the supplementation with oil (I^2^ = 74.1%) and powder (I^2^ = 67.2%) on HbA1c level.

In the meta-analysis of Askari et al. [72], a total of 17 randomised controlled studies were included. A significant association was observed between NS supplementation and reduction in FPG [WMD: −9.93 mg/dL, 95% CI (−13.44, −6.41)], PPBG [WMD: −14.79 mg/dL, 95% CI (−24.19, −5.39)], and HbA1c [WMD: −0.57%, 95% CI (−0.77, −0.37)]. Subgroup analysis revealed that NS oil was more effective than the powder in lowering FPG. Moreover, Bule et al. [7] carried out a systematic review and meta-analysis on the antidiabetic properties of TQ in 18 animal studies. TQ significantly reduced the glucose level with an overall pooled standardised mean difference (SMD) of −9.176 mg/dL (95%CI: −10.759, −7.593) in the streptozotocin (STZ)-induced diabetes model. TQ also had a statistically significant effect on the body weight of diabetic animals with an overall pooled SMD of 4.509 (95%CI: 3.234, 5.784). Moreover, the overall pooled estimate of serum insulin level was significant with an SMD of 1.681 (95%CI: 0.858, 2.503).

Moreover, based on the findings obtained, it was observed that clinical studies were mainly conducted in regions of the Middle East and Indian subcontinent areas including Iran, Egypt, Saudi Arabia, Pakistan, India, and Indonesia. This observation tally with the regions where NS is used traditionally against diabetes as presented in Table 1. Moreover, NS is cultivated mainly in regions of North Africa, Middle Eastern Mediterranean and the southern areas of Asia including Syria, Turkey, India, Pakistan, and Saudi Arabia which tend to explain the reason of clinical studies being restricted to these areas [3]. However, due to the medicinal interest of this plant, its usage and popularity have increased significantly, and now is being marketed and sold by other countries globally in product lines such as nutritional supplements and soft-gel capsules made with the seed and oil [73]. In the United States, the Food and Drug Administration (FDA) classifies NS as generally recognized as safe (GRAS) for use as a spice, natural seasoning, or flavouring. It is also permitted as a component of dietary supplement products, requiring FDA notification and product manufacturing that conforms with dietary supplement current good manufacturing practices (cGMPs) [74].

Additionally, we found that all clinical studies were carried out on the seeds which tend to corroborate with the part used traditionally. In fact, the seed is the main part used from this plant and the reason why NS is termed as “black seed”. It is important to highlight that there exists some confusion regarding the names of NS which is mainly due to the different countries where it is cultivated and used. In English, it is usually referred to as black cumin or black caraway, although it has no relation to the common Cumin or Caraway used as culinary spices. This is the reason why the term “Black seed” is most popular and best describes this plant. In addition, it was observed that the oil has been mostly clinically studied rather than the ingestion of powdered seeds. This is most likely due to the fact that the oil is more concentrated and contains a greater amount of active ingredients and thus is more potent at lower doses compared to powdered seeds. In addition, the oil can be more easily ingested than powdered seeds considering its strong aroma and bitter, pungent flavour.

Furthermore, compared to the NS plant, only one clinical study was conducted on the antidiabetic potential of TQ although the mechanism of action of this compound has been well studied in vitro and in vivo. It is to be noted that besides its antidiabetic effects, TQ has diverse pharmacological properties including antimicrobial, antihistamine, antioxidant effects, immunomodulator, and anticancer properties. Its beneficial effects in managing oxidative stress, immunomodulation, and various types of cancer have been well studied as well as its role in enhancing the immune system by modulation of various inflammatory mediators [75]. Therefore, it is highly recommended that more clinical studies be conducted on TQ as well as studying clinically the use of nanoparticulate drug delivery systems to enhance its bioavailability for optimum biological effects.

## 4. Conclusions

This review provided an insight into the efficacy of NS and its compound TQ in the clinical management of diabetes. The limitation of this project is that a meta-analysis could not be performed due to heterogeneity among studies. Nonetheless, an attempt was made to provide a critical analysis of the effectiveness and safety of NS. From the findings obtained, NS can be considered a highly bioactive medicinal plant. It is highly recommended that a bioproduct be formulated from NS and pharmacologically validated by in vivo and clinical studies. The possibilities of administration of NS at a higher dose should be evaluated which might give a better response than the conventional drugs used as a positive control. Moreover, trials with higher methodological quality with increasing dosage and duration of intervention, and larger sample size need to be conducted. As observed in the present review, only one clinical study was conducted on the compound TQ, therefore need more studies. Future investigations are also necessary to study the mechanism of actions by which TQ exert its therapeutic antidiabetic effects, thereby serving as valuable starting points for drug discovery and development.

## Figures and Tables

**Table 1:** Traditional uses of *Nigella sativa* L. in the management of diabetes.

Plant Part	Country	Method of Preparation	References
Seed	Morocco	-	[8]
Seed	Morocco	Powder ingested with water, 1 teaspoon, orally, once a day	[9]
Seed	Cyprus	Decoction	[10]
Seed	Iraq	Decoction	[11]
Seed	India	Powder ingested with water: Seed of *Cardamine scutata* L., *Carum copticum* L., *Nigella sativa* L., and *Trigonella foenum-graecum* L. are taken in equal amount and powdered. One teaspoonful of powder is taken daily in the morning on empty stomach with the help of luke warm water. It is taken daily for about twenty days	[12]
Leaf, seed, whole plant	Pakistan	Decoction, infusion, powder ingested; whole plant is soaked in water overnight. Take half daily early in the morning. Leaves are boiled in the water to use daily. Take 1 teaspoon seed powder thrice a day	[13]
Seed	Iraq	Mix with honey 1:1 or 2:1, eat 1 tsp/day. Can also be mixed 1:1 with *Trigonella foenum-graecum*	[14]
Seed	Morocco	7 seeds per day	[15]
Seed	Iraq	Hydrodistilled, powder ingested	[16]
Whole plant	Saudi Arabia	-	[17]
Seed	Algeria	Decoction, infusion, powder ingested	[18]
Seed	Iran	Mixed with honey, infusion	[19]
Seed	Iran	Mixed with honey, infusion	[20]
Seed	Eritrea	Seed added in bread or a spoon of powdered seed taken orally before meal	[21]
Seed	Morocco	Powder ingested	[22]
Seed	Algeria	Decoction, powder ingested	[23]
Seed	Saudi Arabia	Eaten raw: 7 seeds are taken daily in the morning.Infusion of powdered seed is prepared by placing it into adequate amount of hot water. One glassful is taken before every meal.	[24]

**Table 3:** Methodological quality scores for the randomised controlled studies using the Jadad scale.

Species	Study	Randomisation	Method of Randomisation	Double Blinding	Method of Blinding	Description of Withdrawal	Total Score
*Nigella sativa* L.	Kooshki et al. [37]	+	−	+	+	−	3
	Darand et al. [42]	+	−	+	+	+	4
	Jangjo-Borazjani et al. [38]	+	−	+	+	+	4
	Rashidmayvan et al. [43]	+	−	+	−	−	2
	Moustafa et al. [33]	+	+	−	−	+	3
	Heshmati et al. [41]	+	+	+	+	+	5
	Kaatabi et al. [30]	+	+	−	−	+	3
	Hosseini et al. [36]	+	−	+	+	+	4
	Mohtashami et al. [39]	+	−	+	−	+	3
	Ansari et al. [34]	+	+	−	−	+	3
	Rachman et al. [31]	+	+	−	−	−	2
	Qidwai et al. [40]	+	−	+	+	−	3
	Ali et al. [32]	+	+	−	−	+	3

## Data Availability

Not applicable.

## Abbreviations

ACC: Acetyl CoA carboxylase; DW: dry weight; FBG: fasting blood glucose; HbA1c: haemoglobin A1C; HOMA-IR: homeostatic model assessment for insulin resistance; HOMA-S: homeostatic model assessment for insulin sensitivity; HOMA-β: homeostatic model assessment for assessment of beta-cell functionality; IV: intravenous; NAFLD: non-alcoholic fatty liver disease; TQ: thymoquinone; TD2: type 2 diabetes; PO: Oral; PPAR-γ: peroxisome proliferator-activated receptor gamma; PPBG: postprandial blood glucose; QUICKI: quantitative insulin-sensitivity check index; WMD: weighted mean difference.
